# GIBA: a clustering tool for detecting protein complexes

**DOI:** 10.1186/1471-2105-10-S6-S11

**Published:** 2009-06-16

**Authors:** Charalampos N Moschopoulos, Georgios A Pavlopoulos, Reinhard Schneider, Spiridon D Likothanassis, Sophia Kossida

**Affiliations:** 1 Pattern Recognition Lab, Department of Computer Engineering & Informatics, University of Patras, Patra, Rio, GR-26500, Greece; 2 Bioinformatics & Medical Informatics Team, Biomedical Research Foundation of the Academy of Athens, Athens, Soranou Efesiou 4, GR-11527, Greece; 3 Bioinformatics/Structural and Computational Biology, European Molecular Biology Laboratory, Heidelberg, Meyerhofstrasse 1, D-69117, Germany

## Abstract

**Background:**

During the last years, high throughput experimental methods have been developed which generate large datasets of protein – protein interactions (PPIs). However, due to the experimental methodologies these datasets contain errors mainly in terms of false positive data sets and reducing therefore the quality of any derived information.

Typically these datasets can be modeled as graphs, where vertices represent proteins and edges the pairwise PPIs, making it easy to apply automated clustering methods to detect protein complexes or other biological significant functional groupings.

**Methods:**

In this paper, a clustering tool, called GIBA (named by the first characters of its developers' nicknames), is presented. GIBA implements a two step procedure to a given dataset of protein-protein interaction data. First, a clustering algorithm is applied to the interaction data, which is then followed by a filtering step to generate the final candidate list of predicted complexes.

**Results:**

The efficiency of GIBA is demonstrated through the analysis of 6 different yeast protein interaction datasets in comparison to four other available algorithms. We compared the results of the different methods by applying five different performance measurement metrices.

Moreover, the parameters of the methods that constitute the filter have been checked on how they affect the final results.

**Conclusion:**

GIBA is an effective and easy to use tool for the detection of protein complexes out of experimentally measured protein – protein interaction networks. The results show that GIBA has superior prediction accuracy than previously published methods.

## Background

Proteomic data and more specifically PPIs data are of great scientific interest through their connection with important cellular functions such as extra and intra cellular signaling, cell communication etc [1]. Moreover, multi protein complexes reveal insights of the functional and topological organization of the protein networks. In the past years, new high throughput methods for identifying pairwise PPIs have been developed that generate enormous datasets. Depending on the method used, different kinds of protein interactions are recorded. This is the reason why there exist differences on the generated datasets from different methods. The most popular ones are yeast two hybrid systems [2], mass spectrometry [1], tandem affinity purification [3], microarrays [4] and phage display [5].

Each method has its strengths and weaknesses; however every method has a certain error rate for the detection of a protein-protein interaction. The main basic errors are under-prediction and over-prediction (false positive) of protein interactions [6]. Besides that, we currently don't know the real "truth" in these datasets, due to the fact that most of the protein complexes are experimentally not yet determined [7].

Usually, the aggregation of the PPIs of an organism is modeled as an undirected graph, symbolized as ***G ***= -(***V***, ***E***), where nodes (***V***) represent the proteins and edges (***E***) the pairwise PPIs. The graph model makes it easy for many computational methods derived from the graph theory to be applied on these noisy datasets to extract functional modules such as protein complexes. The goal of those approaches is to detect highly connected subgraphs which are protein complex candidates.

Each algorithmic strategy relies on a very different approach. The best known one is the Molecular complex detection algorithm (Mcode) [8]. Another algorithm, that has been characterized for its efficiency [9], is the MCL (Markov Clustering) algorithm [10]. Besides that, King *et al *suggested the RNSC algorithm [11] which uses a cost local search algorithm based loosely on a tabu search meta – heuristic. Another algorithm of the local search approach is the Local Clique Merging Algorithm (LCMA) [12] which first locates cliques in a graph and then tries to expand them. Two algorithms that use the hierarchical approach are the Highly Connected Subgraph method (HCS) [13] and the SideS algorithm [14]. The main concept of these methods is the use of numerous graph min cuts until the stopping criterion of each algorithm is satisfied.

In this paper, we have developed a new clustering tool called GIBA that offers the ability to detect important protein modules such as protein complexes. GIBA implements a two step strategy, where in the first one the whole protein – protein interaction graph is divided into clusters and in the second step these clusters are filtered and only the ones considered important are kept. Extensive experiments were performed on 6 different datasets of yeast organism which are either derived from individual experiments (Tong [15], Krogan [16] and Gavin [1,17]) or from online databases (DIP [18] and MIPS [19]). These datasets vary on the number of proteins as well as the number of interactions composing either sparse (Tong dataset) or relatively dense (MIPS and DIP datasets) graphs. Moreover, by using the recorded yeast protein complexes of the MIPS database, we compared the results obtained from GIBA with 4 other algorithms: Mcode, HCS, SideS and RNSC and examined the derived results based on 5 different metrics. Selecting appropriate combinations between clustering algorithms and filtering methods, GIBA proved its superiority compared to the remaining methods. The undertaken experiments and their results are presented in detail in the Results and Discussion section. Finally, an evaluation of the filter methods has been performed to test how these methods affect the final results and to decide, as accurately as possible, the most effective set of filter parameters that produce the best results.

The remaining of the paper is organized as follows: in the next section, we present the algorithms and the filter methods that are hosted in GIBA tool. In Methods section, the properties of GIBA are presented and the evaluation procedure is presented. In Results and Discussion section we performed extensive experiments on datasets with different properties. Results and Discussion section also contains a discussion about the parameters and the methods that compose the filter of GIBA tool and how these approaches affect the final results. Finally, the conclusions of our work are quoted and the main directions for future work are suggested.

## Methods

To identify accurate protein complexes given a protein-protein interaction network, we built a workflow consisting of a two step procedure [20]. Initially, a protein – protein interaction network is clustered by the MCL or the RNSC algorithm and in the second step the results are filtered based either on individual or on a combination of 4 different methods. These are: a) density, b) haircut operation, c) best neighbour and d) cutting edge. This two step approach maintains only those clusters that have high probability to be real biological complexes. A real biological complex can be defined as a set of proteins that are commonly involved in a biological process [21]. A brief description of the algorithms of the first step (MCL and RNSC) and the methods used for the filtering process is given below.

### Description of the MCL algorithm

The MCL algorithm [10] is a fast and scalable unsupervised clustering algorithm based on simulation of stochastic flow in graphs. The MCL algorithm can detect cluster structures in graphs by a mathematical bootstrapping procedure. The process deterministically computes the probabilities of random walks through a graph, and uses two operators transforming one set of probabilities into another. It does so by using the language of stochastic matrices (also called Markov matrices), which capture the mathematical concept of random walks on a graph.

### Description of the RNSC algorithm

The RNSC algorithm [11] searches for a low cost clustering by composing first an initial random clustering, then iteratively moving one node from one cluster to another in a randomized fashion to improve the clustering cost. In order to avoid local minima, RNSC makes diversification moves and performs multiple experiments. Furthermore, it maintains a tabu list that prevents cycling back to a previously explored partitioning. Due to the randomness of the algorithm, different runs on the same input data produce different outputs.

### Description of the cluster density method

Protein complexes correspond to dense subgraphs or even cliques in protein interaction graphs [22]. Therefore, clusters of high density are more likely to correspond to known protein complexes. The density of a subgraph is calculated by the formula below:



where |***E***| is the number of edges and |***V***| the number of vertices of the subgraph.

### Description of the haircut operation method

Haircut operation is a method that detects and excludes vertices with low degree of connectivity from the potential cluster that these nodes belong to. Proportionally, the lower the connectivity of a node is, the lower the probability for this node to belong to a protein complex is. In such a way, the deletion of such nodes that add noise to the cluster leads to protein complexes that are more likely to be present in nature.

### Description of the best neighbour method

In contrast with haircut operation method, best neighbour method tends to detect and enrich the clusters with candidate vertices that are considered as good "neighbours". Such a node is the one where the proportion of its edges adjacent to the cluster divided by the total degree of the vertex is above a threshold defined by the user:



The best neighbor method is mostly suitable to detect larger protein complexes that offer extra information about protein complexes included in a protein interaction dataset. Another advantage of using best neighbor method is that a protein can be assigned to more than one protein complex as it is known that there are shared components between protein complexes.

### Description of the cutting edge method

Analyzing the structure of a protein-protein interaction network, molecular modules are densely connected within themselves but are sparsely connected to the rest of the network [23]. To address these cases, a filtering criterion was applied, called cutting edge and is defined as:



where |***inside edges***| is the number of edges inside a cluster and |***total edges***| is the number of edges that are adjacent to at least one vertex of the cluster. The clusters in which the cutting edge metric is below a user defined threshold are discarded from the filter of our method.

### Evaluation procedure

In order to test the efficiency of GIBA, we have compared it with 4 other algorithmic methods: the Mcode, the HCS, the SideS and the RNSC algorithm as it was presented in [11]. The benchmark that we have used to evaluate the algorithms tested consists of known yeast protein complexes retrieved from the MIPS database. MIPS protein complexes composed from smaller ones, also recorded in MIPS database, were removed to avoid redundancy. The final evaluation dataset comprises 220 complexes.

In addition to the collection of MIPS protein complexes, we have also used the same evaluation metric adopted in [8], called geometric similarity index. This method considers a predicted complex as valid if  where **I **is the number of common proteins, **A **the number of proteins in the predicted complex and **B **the number of proteins in the recorded complex. In our measurements, we have calculated the mean geometric similarity index of the valid predicted complexes called mean score.

Furthermore, 4 different matching statistic metrics, that were presented in [9], were used in the evaluation process of the algorithms tested. These are *sensitivity *(Sn), *Positive Predictive Value *(PPV) and *Geometrical Accuracy *(Acc_g). These metrics are typically used to measure the correspondence between the result of a classification and a reference. Sensitivity is defined as the fraction of proteins of a recorded protein complex in MIPS database that are found in a cluster. Positive predictive value is the proportion of members of a cluster which belong to a recorded complex, relative to the same number of members found in all recorded complexes. The geometrical accuracy is measured through the geometrical mean of the sensitivity and the positive predictive value. It has the advantage that gives a more "objective" picture of the quality of the results as it obtains high values only if the values of sensitivity and of positive predictive value metrics are high.

### Datasets

To demonstrate the use of our methodology, we have used six datasets derived from various small scale and high-throughput methods. The multifaceted nature of the datasets enables us to perform a more "objective" comparison of the algorithms tested. In this section, we give a short description of the datasets that were used.

#### Tong dataset

This network consists of 7430 edges and 2262 vertices [15]. A genetic interaction network was mapped by crossing mutations in several genes into a set of viable gene yeast deletion mutants scoring the double mutant progeny for fitness defects. The interactions of this network were produced by predicting the functions of the interactive elements often produced by bringing together functionally related genes or components or elements that belong to the same pathway. The genetic network exhibited dense local neighbourhoods; our method aims to go one step further by predicting these neighbourhoods but also by splitting them in smaller groups that are functionally more significant.

#### Krogan dataset

This dataset consists of 7088 edges and 2675 vertices and contains different tagged proteins of the yeast *Saccharomyces cerevisiae*. In a previous analysis [16], the MCL algorithm was used to cluster and organize the proteins into several groups so that about half of them were absent from the MIPS database. We observed that a small amount of noise was added to these data and therefore we have applied our method to detect and filter the groups detected by MCL.

#### Gavin_2002–2006 datasets

In this case, we have used two networks, the first consisting of 3210 edges and 1352 vertices and the second consisting of 6531 edges and 1430 vertices [1,17]. In the first dataset, large-scale tandem affinity purification and mass spectrometry were used to characterize multiprotein complexes in *Saccharomyces cerevisiae*. Extending this information to human genome, this dataset provides an outline of the eukaryotic proteome as a network of protein complexes. Using the whole network, we try to see how successfully our method isolates the network complexes. The second dataset comes with the first genome-wide screen for complexes in yeast.

#### DIP dataset

The Database of Interacting Proteins (DIP) is a database that documents experimentally determined protein-protein interactions [18]. We have used this database to isolate a network consisting of 17491 edges and 4934 vertices. One of the reasons why we have included this source data for our experiments is that beyond cataloging details of protein-protein interactions, the DIP database helps us not only to understand protein functions but also the value of protein-protein relationships as well. The used DIP dataset version in our experiments was the one of 04/03/2007.

#### MIPS dataset

The Munich Information Center for Protein Sequences provides resources mainly related to genome information [19]. Most of the databases that contain information about a variety of genomes of different organisms are manually curated. Furthermore 400 genomes that were automatically annotated are also included. One of the aims of this database is to provide information related to interactions such as PPIs. In this study case, we have isolated a network consisting of 12526 edges and 4554 vertices given by the MIPS database. The used MIPS dataset in our experiments was created on 05/18/2006.

### Implementation

The GIBA tool is a java application, while the RNSC, MCL and the methods used in the filtering process are implemented in C language. Three out of the four algorithms that were used in our experiments (SideS, RNSC and HCS) were implemented in C language too. The Mcode algorithm is implemented as a java plugin for Cytoscape [24]. All the experiments were performed using an Intel Double Core 2.13 GHz processor, with 2 GB of RAM and Microsoft windows XP. Loop edges were not taken into account.

The filter we have used for the results of the RNSC algorithm was composed by two out of three parameters as they are presented in [11] (size and density). We did not use the third parameter (functional homogeneity) as this kind of information was not available for all datasets so that the comparison with the other algorithms, which did not use this kind of information, would not be biased. The SideS and HCS algorithms do not take any parameters, whereas for the use of Mcode and MCL algorithms we used the optimal parameters for accuracy as they are defined in [9].

## Results and discussion

### The GIBA tool

**Figure 1:**
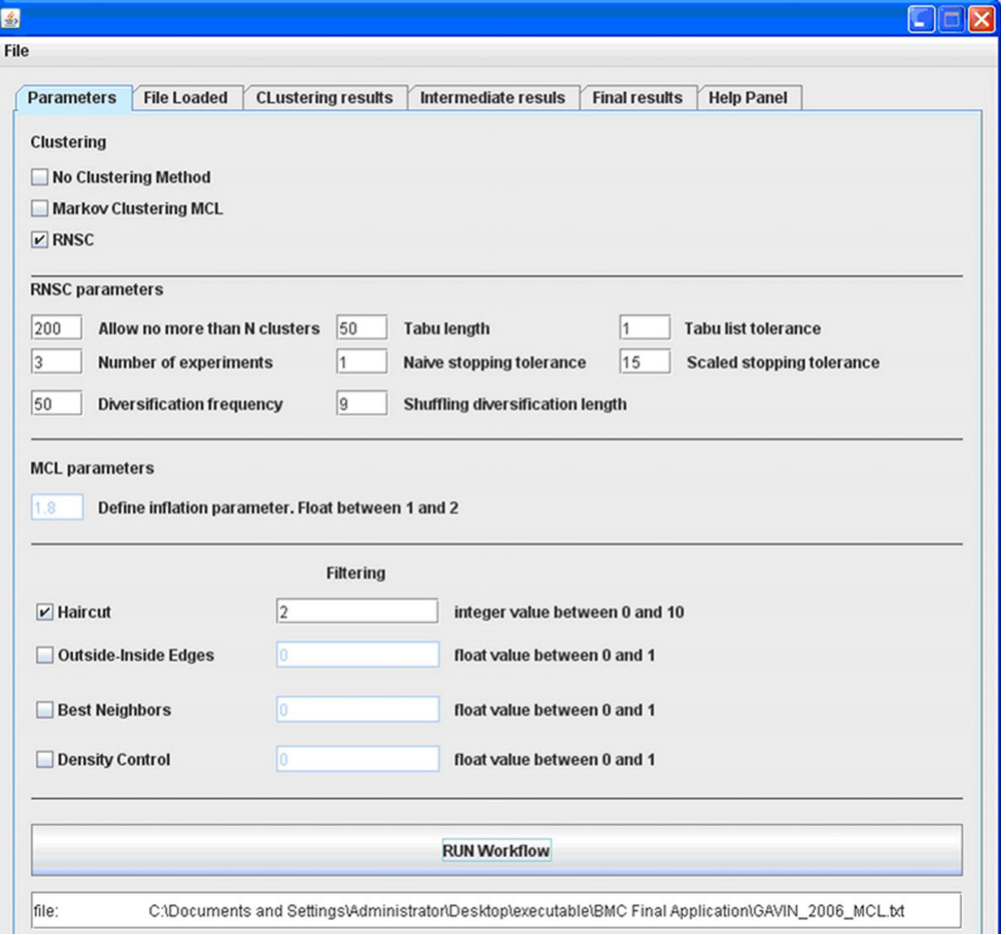
**The main window of the GIBA tool**.

GIBA provides an extremely user-friendly environment. Users without informatics background could perform clustering without any difficulties. Figure 1 shows the main window of the GIBA tool.

The workflow of the tool is straightforward. Initially, the user loads a tab delimited file that contains a simple weighted list of the protein – protein interactions. Then, the user can choose either the MCL or the RNSC algorithm and define their parameters to cluster the protein interaction network. Initially, the parameters of each algorithm have default values which offer the maximum accuracy according to [9]. In the third step, the user chooses which methods will constitute the filter and defines the necessary parameters. Depending on the selections of the algorithm and methods, the corresponding parameters are set to active state, while all the others are set to inactive state. Moreover, there are pop up error messages that inform the user for potentially wrong parameter values. Finally, the user can press the "Run Workflow" button and start the clustering process. The Help Panel of GIBA is also providing explanations about the algorithms incorporated and their parameters.

After a successful run, GIBA generates various outputs: The "File loaded" tag shows the contents of the input file that is the protein – protein interactions. The proteins that constitute the clusters which derived from the first step clustering (the MCL or RNSC algorithm results) are shown in the "Clustering results" tag, while the interactions into each cluster are presented in the "Intermediate results" tag. Every file is stored locally on the hard disk so the user can reuse the intermediate results by skipping the time consuming run of MCL or RNSC algorithm. The final results, after the filtering, are presented on the "Final results" tag, where the number and the labels of the proteins that constitute the final clusters are shown. In addition, the number of interactions for each final cluster is also presented. This file is also stored on the local hard disk drive.

Many screen shots of the use of GIBA as well as information about GIBA algorithms and methods are given in Additional File 1.

### Comparison with other algorithms

We have compared GIBA results with those derived from 4 different algorithms: Mcode, SideS, HCS and RNSC as it has been presented in [11]. All the results of our experiments are presented in Additional File 2.

**Figure 2:**
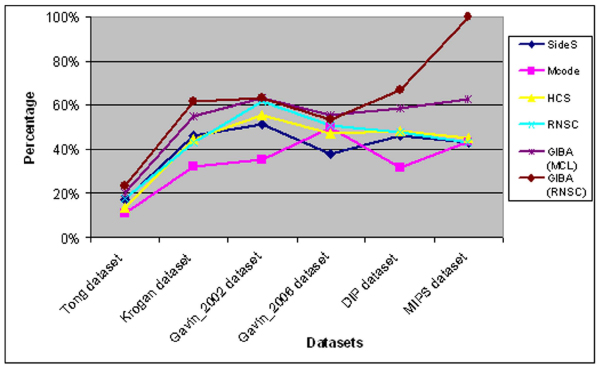
**The percentage of successful predictions in respect to the MIPS recorded complexes of the algorithms tested**.

GIBA produces better results than the aforementioned methods either by selecting the MCL or the RNSC algorithm. These results prove the efficiency of the second algorithmic step followed by GIBA as they are even better from those obtained from the filter process used in RNSC in [11]. In order to obtain and compare these results, we have applied the same filter options on both algorithms used in the first step of GIBA function in our experiments (MCL and RNSC). In figure 2, the percentage of successful predictions of every algorithm on each dataset is presented. In the first dataset (Tong) the algorithms tested could not identify many real protein complexes as these datasets are extremely sparse and full of noise. However, even on this dataset, the algorithmic strategies offered by GIBA obtain the higher accuracy. When GIBA uses RNSC as a first step, it achieves exceptionally good results for the online databases datasets (MIPS and DIP dataset). This happens due to the fact that very few clusters are produced in those cases. Nevertheless, when GIBA uses MCL, it may produce fewer clusters. However the difference between the absolute number of clusters produced by GIBA and the other algorithms tested is not so big.

**Figure 3:**
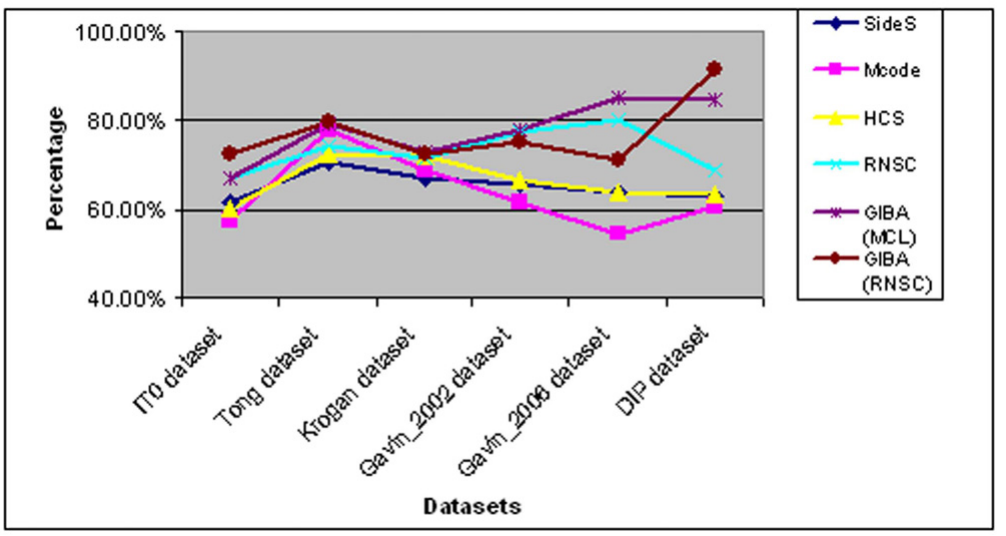
**The performance of the algorithms concerning Acc_g metric**.

In figure 3, the performance of the algorithms is presented concerning the geometrical accuracy metric. As we mentioned before, geometrical accuracy offers a better insight about the quality of the results of each algorithm. Even with small differences as it can be seen in Additional File 2, the GIBA algorithmic approaches achieve better results. In most of the cases, GIBA with the use of MCL algorithm produces better quality results than those produced by the use of RNSC algorithm. On the other hand, GIBA with RNSC has achieved better rate of successful predictions of protein complexes. Moreover, the mean score of the valid predicted complexes shows that GIBA results in much better approximations of the recorded protein complexes. The older version of RNSC algorithm produces good approximations too, but comparing to GIBA approaches, it achieves poorer results on the geometrical accuracy and on the percentage of successful predictions.

The use of the methods that compose the filtering process was in each case different and dependent on the nature of the input protein – protein interaction graph. We tried to achieve good prediction rate without minimizing the number of the final GIBA clusters that will pass the filtering process. In sparse graphs, better results were obtained without the need of the methods of best neighbor and cutting edge, while the methods of density and haircut operation affect vastly the final results. Table 1 shows the methods and their parameters used in the filtering process in each case.

**Table 1:** The methods used in the filtering process.

**Dataset**	**Filter**
**Tong**	Density = 0.75, Haircut = 2
**Krogan**	Cutting_Edge = 0.55, Density = 0.7, Haircut = 3
**Gavin_2002**	Cutting_Edge = 0.5, Density = 0.6, Haircut = 2
**Gavin_2006**	Cutting_Edge = 0.75, Density = 0.6, Haircut = 2, Best_neighbor = 0,6
**DIP**	Cutting_Edge = 0.5, Density = 0.6, Haircut = 3
**MIPS**	Cutting_Edge = 0.5, Density = 0.7, Haircut = 2, Best_neighbor = 0,75

### Analysis of GIBA filtering methods

As it was proved, the GIBA results are sensitive to the methods and their parameters that were used in the filtering process. Therefore, we have tested the possible combinations of the 4 methods that compose the filtering process in order to see how they affect the final results and how the function of one method affects the others.

We have chosen specific range of values for each method parameter:

• for the density parameter: **[*0.55, 0.8*]**

• for the haircut operation parameter: ***2 or 3***

• for the best neighbor parameter: **[*0.6, 0.75*] **and

• for the cutting edge parameter: **[*0.6, 0.75*]**.

Choosing a parameter value out of the proposed range would be meaningless because the parameter method would become either too rigorous and it would produce very few clusters (if it was higher than the proposed maximum) or would add noise to the final data (if it was lower than the proposed minimum).

We have examined all possible combinations, using a parameter step of 0.5, in three datasets with different properties: 2 online database dataset (MIPS and DIP) and a dataset from individual experiment (Gavin_2006). So we have run 192 different filter combinations for each dataset.

**Figure 4:**
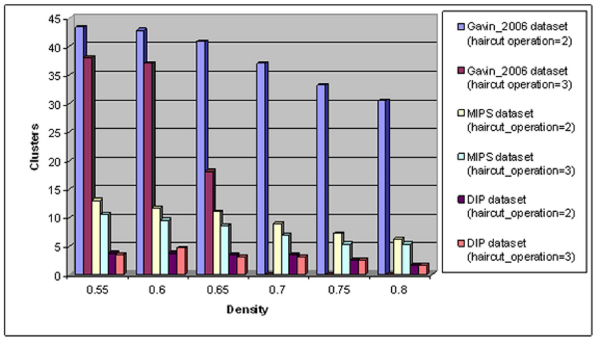
**Impact of density and haircut operation parameters to the produced number of clusters**.

From our experiments it was shown that the most important method affecting most the final results is the density method. In figure 4, we present the impact of density and haircut operation to the produced number of GIBA clusters. For each density parameter value, we calculate the mean number of clusters, produced by 16 different parameter combinations (regarding the best neighbor and cutting edge parameter). As it can easily be seen, the number of the final cluster is reduced when the value of density or haircut operation parameter rises. More specifically, in Gavin_2006 dataset, no clusters are produced when haircut operation parameter has value equal to 3 and density parameter is equal to 0.7 or more.

**Figure 5:**
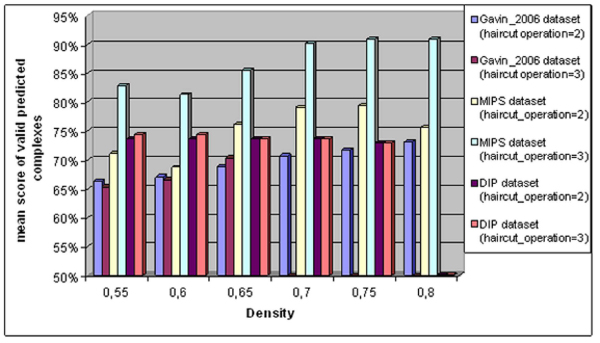
**Impact of density and haircut operation parameters to the mean score of valid predicted complexes**.

However, the increase of density parameter improves the quality of the identification of protein complexes. As it can be seen in figure 5, there is a slight improvement on the mean score due to the increase of the density parameter. Moreover, the increase of the haircut operation parameter does not offer any benefits.

**Figure 6:**
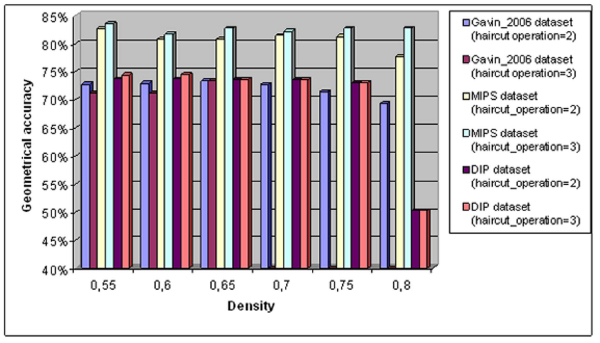
**Impact of density and haircut operation parameters to geometrical accuracy metric**.

Although, the increase of density causes positive effect on mean score metric, we could not claim the same thing for the geometrical accuracy metric. In figure 6, it is shown that this metric is slightly reduced when density or haircut operation parameter increases. Because of the high values of density parameter, smaller clusters are produced at the end of the procedure. The proteins that constitute a cluster may be reduced even more if the haircut operation parameter has also a high value. Small clusters can not achieve high scores in sensitivity and positive predictive value metric because of the definition of these metrics. Further research is needed, using intelligent techniques in order to predict the optimal value of the other parameters.

## Conclusion and future work

In this paper, we have introduced the GIBA tool in order to identify protein complexes from pairwise protein – protein interaction datasets. GIBA workflow splits in a two step process: initially, it clusters the whole input protein network and afterwards it applies a filtering process to obtain the final clusters. In addition, GIBA is user friendly and can also provide intermediate results for every step that can be useful for further use. With our experiments we proved the efficiency of GIBA comparing to 4 other methods.

The main issue of our future work will be the appliance of machine learning methods to detect how the properties of the initial protein – protein interaction dataset can take advantage of the filtering process in order to achieve better results. This could lead to the development of a new algorithmic approach with adaptive behavior relative to the initial protein network.

## Availability and requirements

1. Project name: GIBA: A clustering tool for detecting protein complexes

2. Project home page: 

3. Operating system(s): Windows.

4. Programming language: Java and C.

5. License: GNU GPL.

6. Any restrictions to use by non-academics: No.

## Competing interests

The authors declare that they have no competing interests.

## Authors' contributions

CNM conceived and developed the idea of the GIBA tool to perform clustering on PPI graphs. CNM also wrote the code for the filtering methods, performed the experiments and wrote the manuscript. GAP implemented the GIBA tool as java application, wrote part of the manuscript and revised the manuscript. RS made contribution to the experiments and revised the manuscript. SDL was responsible for the experiments performed; the metrics used in their evaluation and revised the manuscript. SK directed the whole project, wrote and revised the manuscript.

## Supplementary Material

Additional file 1 Guide to use GIBA tool. Click here for file

Additional file 2 **Summary of experimental results**. The percentage of successful predictions is shown in the first column; the absolute number of valid predicted complexes is shown in the second column as well as the total number of predicted complexes. The mean score of the valid predicted complexes is shown in the third column. The last three columns present the Sensitivity (Sn), the Positive Predictive Value (PPV) and the geometric Accuracy (Acc_g) respectively. Click here for file
