# Supplementary material for: GIBA: a clustering tool for detecting protein complexes
Source: BMC Bioinformatics. 2009 Jun 16;10(Suppl 6):S11. doi: 10.1186/1471-2105-10-S6-S11 (PMC2697634; doi:10.1186/1471-2105-10-S6-S11)
Supplement: Additional file 1 — Guide to use GIBA tool. [file 1471-2105-10-S6-S11-S1.doc]

**INSTALLATION**

------------------------------------------------------------------------------------------------------------

This program is available only for windows.

JAVA 6 or higher should be preinstalled for the application to run.

The files that are needed are: BIBE2008.exe, rnsc.exe, cygwin1.dll and Enhanced_MCL.jar

To run from command line just place all of the files in the same folder and type

java -Xmx350M -jar 'jar filename'

The parameter -XMX defines the size of the memory - in this example 350MB of RAM

For more questions do not hesitate to send an e-mail in the addresses below:

[cmoschop@bioacademy.gr](mailto:cmoschop@bioacademy.gr)

or

[pavlopou@embl.de](mailto:pavlopou@embl.de)


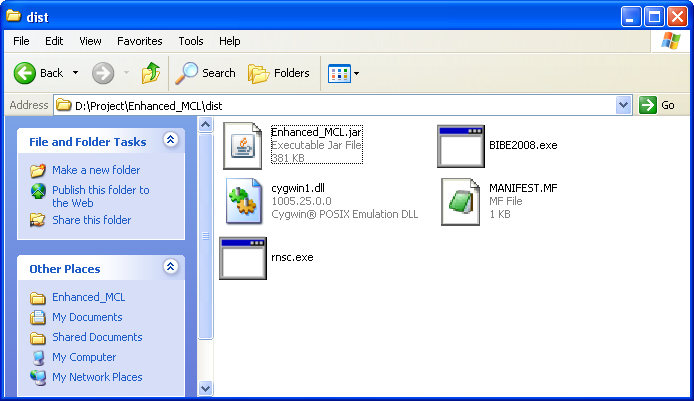


**GENERAL INTERFACE**

**This is the main panel with the default parameters for the users.**


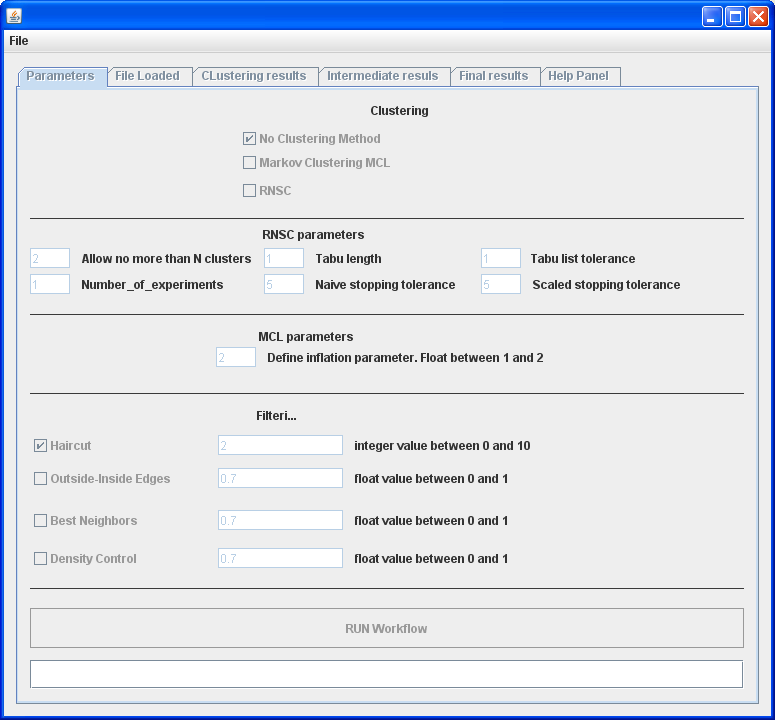


PARAMETERS - RNSC

------------------------------------------------------------------------------------------------------------

-Allow no more than N clusters

Allow no more than "num" clusters. "num" must be between 2 and n,

where n is the number of vertices in the graph. If the -c option is

not specified or an invalid value is given, n clusters are used.

-Tabu length

Set the tabu length to "num". Default value is 1. Note that when the

-T option is used, vertices can appear on the tabu list more than once

and moving them is only forbidden when they are on the tabu list more

than TabuTol times, where TabuTol is the tabu list tolerance.

-Tabu list tolerance

Set the tabu list tolerance to "num". Default value is 1. The tabu

list tolerance is the number of times a vertex must appear on the tabu

list before moving it is forbidden.

-n num

Set the naive stopping tolerance to "num". Default value is 5. This

is the number of steps that the naive scheme will continue without

improving the best cost. If you run the scaled scheme, using a higher

naive stopping tolerance, it is not likely to improve your results.

-N num

Set the scaled stopping tolerance to "num". Default value is 5. This

is the number of steps that the scaled scheme will continue without

improving the best cost. Setting the tolerance to 0 will cause the

algorithm to skip the scaled scheme.

-Number_of_experiments

Run "num" experiments. The best final clustering over all experiments

will be written to file. Default is 1.

MCL PARAMETER

--------------------------------------------------------------------------------------------------------

MarkovClustering implements the Markov clustering (MCL) algorithm for graphs, using a HashMap-based sparse representation of a Markov matrix, i.e., an adjacency matrix m that is normalised to one. Elements in a column / node can be interpreted as decision probabilities of a random walker being at that node. Note: whereas we explain the algorithms with columns, the actual implementation works with rows because the used sparse matrix has row-major format.

The basic idea underlying the MCL algorithm is that dense regions in sparse graphs correspond with regions in which the number of k-length paths is relatively large, for small k in N, which corresponds to multiplying probabilities in the matrix appropriately. Random walks of length k have higher probability (product) for paths with beginning and ending in the same dense region than for other paths.

The algorithm starts by creating a Markov matrix from the graph, where first, in the adjacency matrix, diagonal elements are added to include self-loops for all nodes, i.e., probabilities that the random walker stays at a particular node. After this initialisation, the algorithm works by alternating two operations, expansion and inflation, iteratively recomputing the set of transition probabilities. The expansion step corresponds to matrix

multiplication (on stochastic matrices), the inflation step corresponds with a parametrized inflation operator Gamma_r, which acts column-wise on (column) stochastic matrices (here, we use row-wise operation, which is analogous).

The inflation operator transforms a stochastic matrix into another one by raising each element to a positive power p and re-normalising columns to keep the matrix stochastic. The effect is that larger probabilities in each column are emphasized and smaller ones deemphasized. On the other side, the matrix multiplication in the expansion step creates new non-zero elements, i.e., edges. The algorithm converges very fast, and the result is an idempotent Markov matrix, M = M * M, which represents a hard clustering of the graph into components.

Expansion and inflation have two opposing effects: While expansion flattens the stochastic distributions in the columns and thus causes paths of a random walker to become more evenly spread, inflation contracts them to favoured paths.

Description is based on the introduction of Stijn van Dongen's thesis Graph Clustering by Flow Simulation (2000); for a mathematical treatment of the algorithm and the associated MCL process, see there.

@author gregor :: arbylon . net

OTHER PARAMETERS

-----------------------------------------------------------------------------------------------

Cluster Density

The density of a subgraph is calculated by the formula below:

2|E|/(|V|*(|V|-1)),

where |E| is the number of edges and |V| the number of vertices of the subgraph.

Haircut operation

Haircut operation is a method that detects and excludes vertices with low degree of connectivity from the potential cluster that these nodes belong to. Proportionally, the lower the connectivity of a node is, the lower the probability for this node to belong to a protein complex is. In such a way, the deletion of such nodes that add noise to the cluster leads to protein complexes that are more likely to be present in nature.

Best neighbor method

In contrast with haircut operation method, best neighbor method tends to detect and enrich the clusters with candidate vertices that are considered as good "neighbors". Such a node is the one where the proportion of its edges adjacent to the cluster divided by the total degree of the vertex is above a threshold defined by the user:

(adjacent edges/total edges) >threshold

The best neighbor method is mostly suitable to detect larger protein complexes that offer extra information about protein complexes included in a protein interaction dataset.

Cutting edge metric

Analyzing the structure of a protein–protein interaction network, molecular modules are densely connected within themselves but are sparsely connected to the rest of the network. To address these cases, a filtering criterion was applied, called cutting edge and is defined as:

|inside edges|/|total edges|

where |inside edges| is the number of edges inside a cluster and |total edges| is the number of edges that are adjacent to at least one vertex of the cluster. The clusters in which the cutting edge metric is below a user defined threshold are discarded from the filter of our method.

**INPUT FILE**

**The input file is usually a list of weighted or unweighted connections. An example is given below.**

**cat hat 1**

**hat bat 1**

**bat cat 1**

**bit fit 1**

**fit hit 1**

**hit bit 1**

**or**

**RPL7A IPI1 1**

**EFT2 ACS2 1**

**ZUO1 ASC1 1**

**NAB3 NAB6 1**

**MED7 YAP1 1**

**NOC4 IMP3 1**

**MRPL3 MRP7 1**

**MRP7 MRPL7 1**

**NOP13 ERB1 1**

**FUR1 RVB2 1**

**TPS1 IML1 1**

**IML1 CTR9 1**

**RPN2 SAM1 1**

**RPL13A BRX1 1**

**SEC27 RVB2 1**

**RSC8 HHF2 1**

**RPL2A RPL25 1**

**YMR31 CDC19 1**

**SSN2 MED6 1**

**RAD50 RPT3 1**

**APL6 HMO1 1**

**RIX7 RRP1 1**

**ERG13 RPS25A 1**

**ERG13 RRN3 1**

**WTM1 ARO3 1**

**The file should be TAB DELIMITED**

**USAGE**

**Load a file with connections like here,**

**
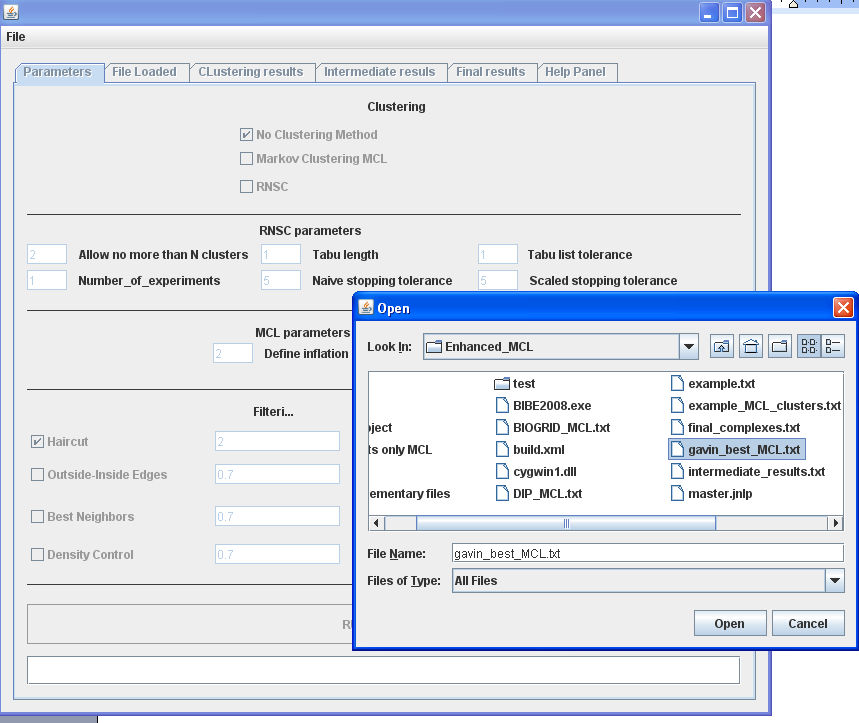
**

**Select and parameterize the algorithms after loading. You can chose MCL or RNSC and then filter the results using one of the secondary methods,**

**
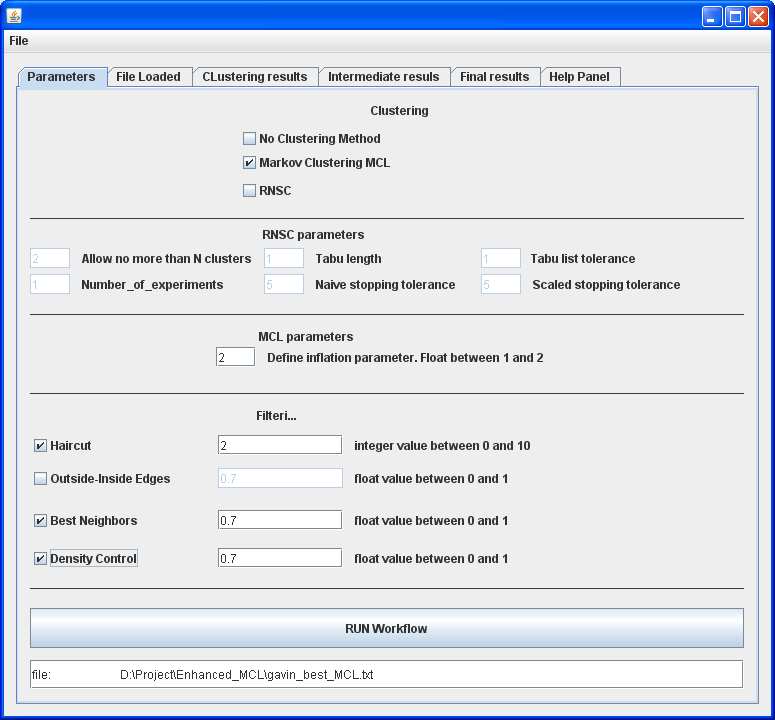
**

**
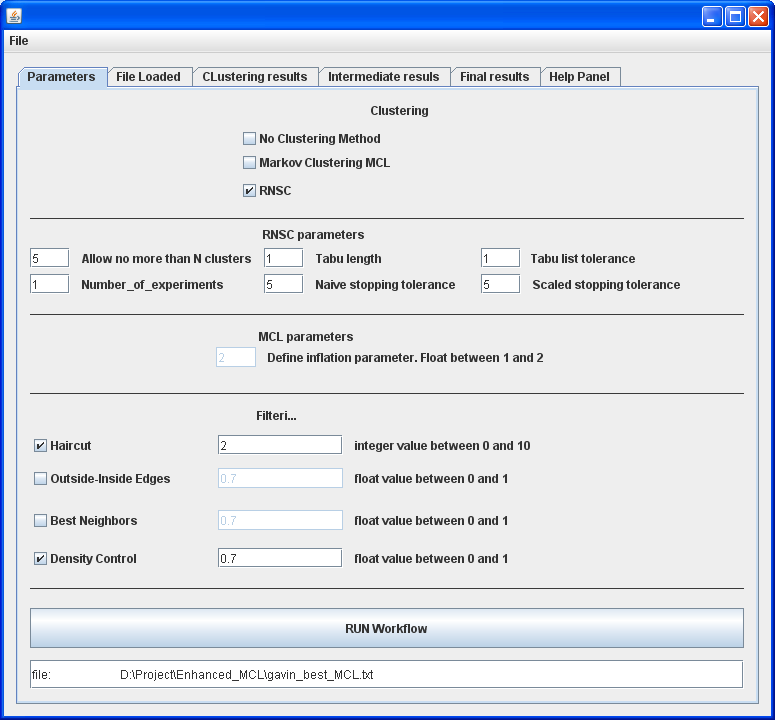
**

**And then click the run button to run the workflow.**

**
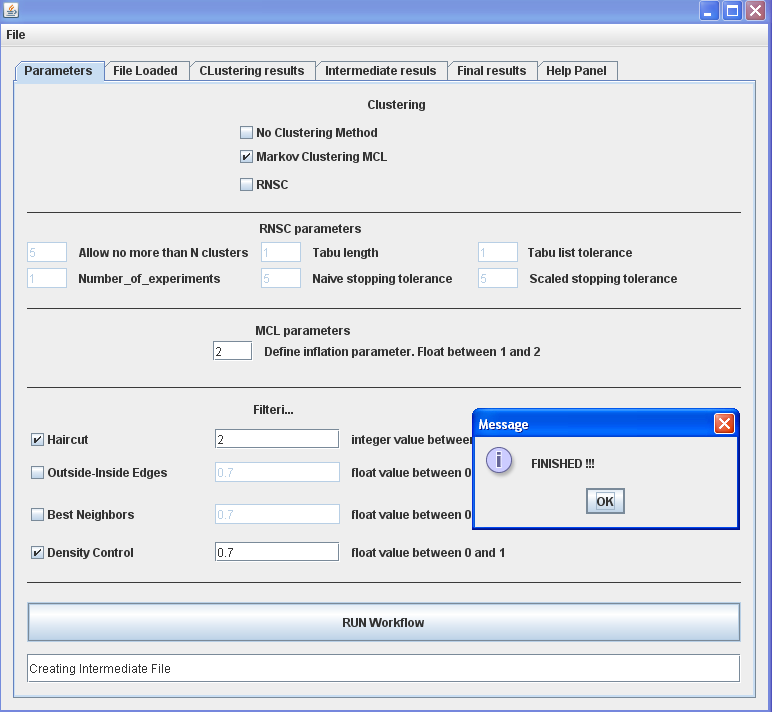
**

**This Panel shows the contents of the file that was loaded,**

**
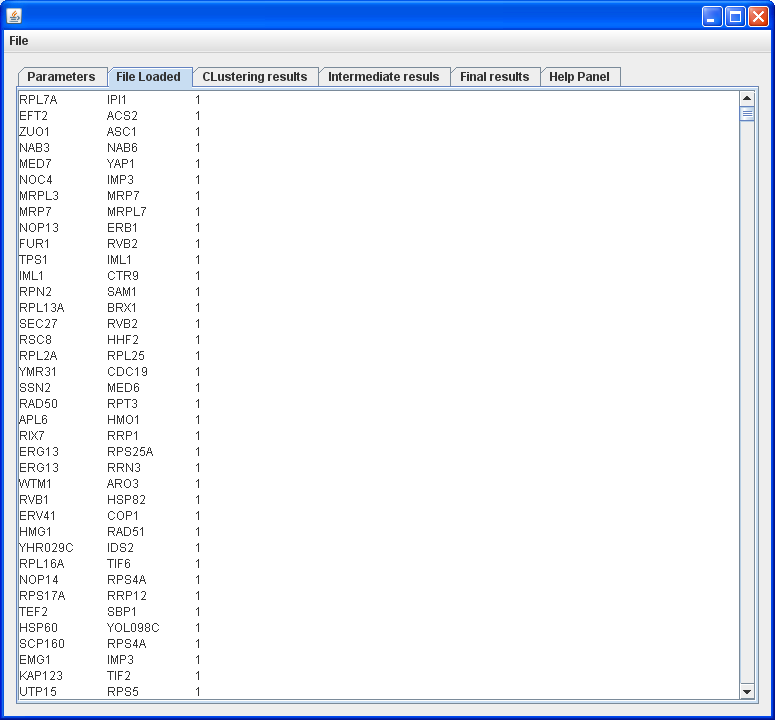
**

**This Panel shows the results of the clustering that were produces by the initial methods RNSC or MCL.**

**
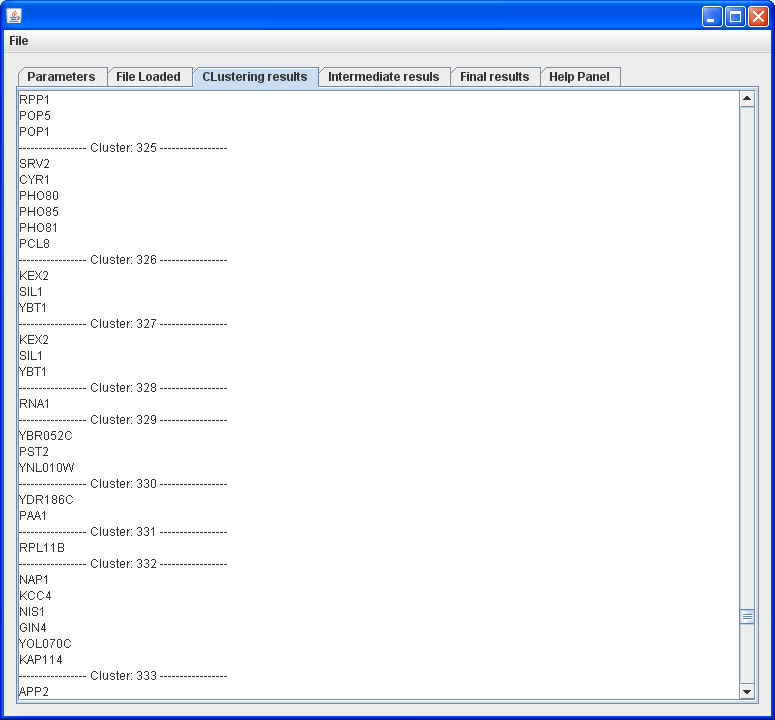
**

**This Panel shows the intermediate results produced by the clustering methods.**

**This will be later the input for the secondary methods.**

**This file like every file is stored locally on the hard disk. This is because in the case of a big dataset, someone can load this file directly by skipping the time consuming run of MCL. This was done mainly for time efficiency purposes.**

**
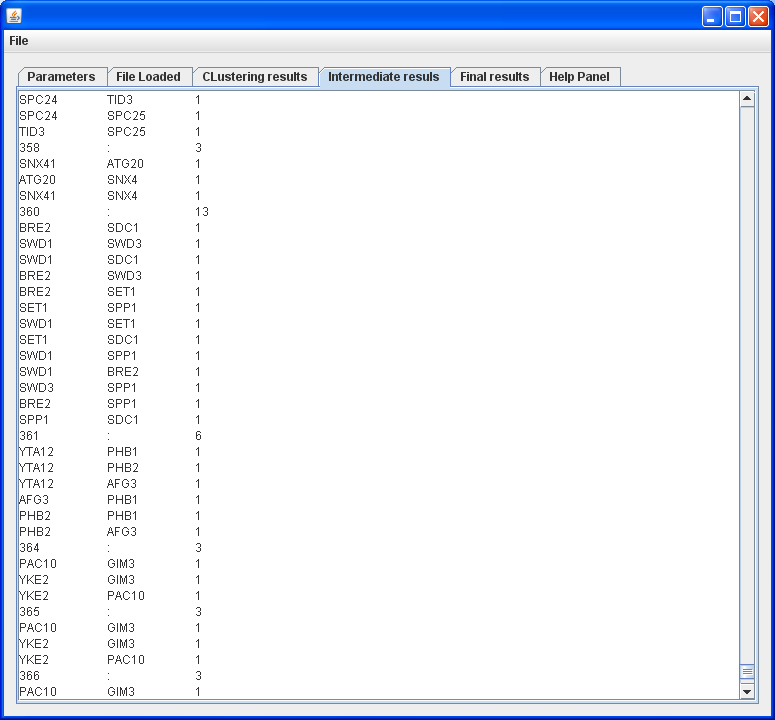
**

**This panel which is the most important one shows the results of the workflow.**

**Here we can see the number of clusters together with their elements. Results are also stored locally on the hard disk drive.**

**
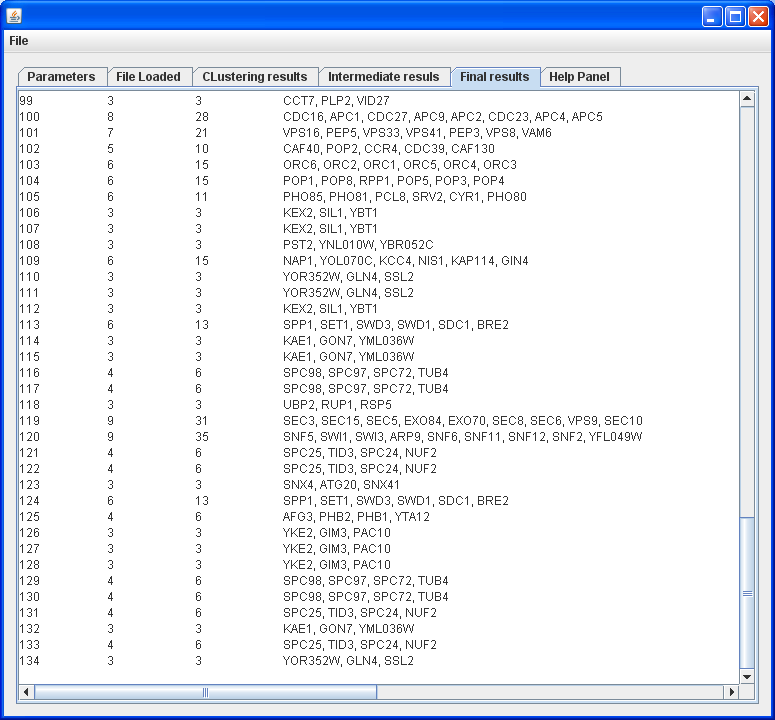
**

**This is the help panel where someone can see the meaning of the various parameters of the algorithms.**

**
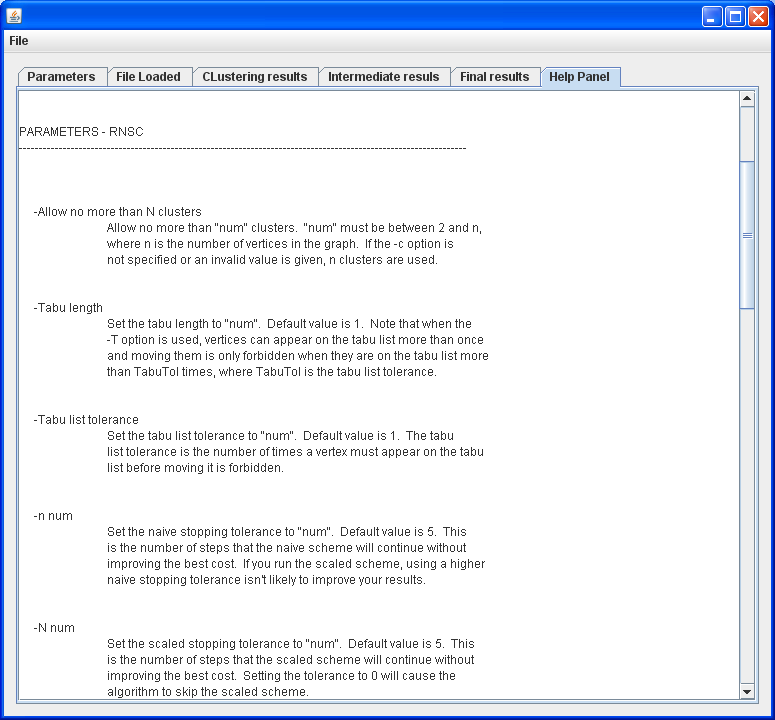
**
